# Fat Modulates the Relationship between Sarcopenia and Physical Function in Nonobese Older Adults

**DOI:** 10.1155/2012/216185

**Published:** 2012-01-23

**Authors:** Robin L. Marcus, Diana I. Brixner, Sameer Ghate, Paul LaStayo

**Affiliations:** ^1^Department of Physical Therapy, University of Utah, Salt Lake City, UT 84108, USA; ^2^Department of Pharmacotherapy, University of Utah, Salt Lake City, UT 84108, USA

## Abstract

It is intuitive to think that sarcopenia should be associated with declines in physical function though recent evidence questions this assertion. This study investigated the relationship between absolute and relative sarcopenia, with physical performance in 202 nonobese (mean BMI = 26.6 kg/ht^2^) community-dwelling older (mean age = 73.8 ± 5.9 years) adults. While absolute sarcopenia (appendicular skeletal mass (ASM)/ht^2^) was either not associated, or weakly associated with physical performance, relative sarcopenia (ASM/kg) demonstrated moderate (r = 0.31 to r = 0.51, *P* < 0.01) relationships with performance outcomes in both males and females. Knee extension strength (r = 0.27) and leg extension power (r = 0.41) were both related to absolute sarcopenia (*P* < 0.001) in females and not in males. Strength and power were associated with relative sarcopenia in both sexes (from r = 0.47 to r = 0.67, *P* < 0.001). The ratio of lean mass to total body mass, that is, relative sarcopenia, is an important consideration relative to physical function in older adults even in the absence of obesity. Stratifying these individuals into equal tertiles of total body fat revealed a trend of diminished regression coefficients across each incrementally higher fat grouping for performance measures, providing further evidence that total body fat modulates the relationship between sarcopenia and physical function.

## 1. Introduction

It seems intuitive that muscle structure (lean) and function (strength and power) are intricately linked to an older individual's level of physical function. Moreover, sarcopenia or the age-associated loss of lean tissue, along with increased whole-body and regional fat deposits, is a normal manifestation of old age [1]. There is substantial debate, however, as to whether sarcopenia can explain the age-diminished levels of physical function in older individuals. A recent review of the literature [1] highlights trends demonstrating that high whole-body fat mass (obesity) is more influential than low whole-body lean mass with respect to mobility and functional decline. The current consensus statement from the European Working Group on Sarcopenia in Older People [2], not specifically highlighting obesity per se, also reinforces the notion that lean body mass alone is not adequate to identify functional decline. Further, because absolute sarcopenia, defined as the ratio of appendicular skeletal mass (ASM) to an individual's height squared (ASM/ht^2^), does not demonstrate strong associations with physical function [3], it follows that lean body mass only weakly explains the lower levels of physical functioning in older adults.

 While this uncoupling of lean body mass and physical functioning may not be surprising in obese individuals, it remains counterintuitive in those older adults who are not obese. However, decreased activity and illness, which commonly occur even in nonobese older adults, may result in substantial loss of lean tissue mass while body fat remains relatively constant or perhaps increased. It is likely, therefore, that the traditional BMI derived definition of obesity (BMI ≥ 30 kg/m^2^) does not accurately identify abnormal body composition in older adults, who in particular are experiencing a dual loss of lean tissue and an increase in adiposity, even when their BMI falls below the obese level. Therefore, the purpose of this paper is to determine in nonobese older adults whether total body adiposity modulates the relationship between sarcopenia and physical function.

## 2. Methods

### 2.1. Subjects

Participants eligible for this study were recruited from an ongoing study via a large database of the Utah Health Research Network from July 2007 to June 2009. The recruitment strategy for the ongoing study utilized an opt-out mailing system that required potential subjects not interested in participating to call the study coordinator to opt-out within 5 business days of receiving the recruitment letter. Subjects who did not opt out were contacted via phone by a research assistant 10 days after receiving the letter. Subjects were briefed about the study, their eligibility was confirmed, and they were verbally consented to participate. Those interested in participating in the study were scheduled for a dual X-ray absorptiometry (DXA) scan and the physical function tests on two separate days. A research assistant met subjects at the first of these two visits and administered the written informed consent for study participation. The inclusion criteria were age greater than 64 years, male or female, total weight < 300 pounds, height < 6′5′′ (maximum table weight and height for DXA), and a willingness to come to the clinic for DXA and for physical function testing. Subjects were excluded from participation if they had tests with radiographic contrast material in the past 72 hours, had participated in nuclear medicine studies in the past 3 days, or were unable to independently ambulate.

### 2.2. Procedures

Participants underwent whole-body DXA to provide estimates of lean and fat tissue mass (DXA, Hologic QDR QDR-4500A; Waltham, MA) using the standardized procedures of the manufacturer (software version 12.6.6 : 7). Parameters for analysis were followed as identified in the user's guide (QDR Series User's Guide, March 2000). A quality control system has been established to optimize the accuracy of the data obtained from the DXA. Calibration of the DXA is confirmed weekly through spine phantom scans. Algorithms are in place to detect drift and suboptimal performance of the machine, and no long-term bias was evident in the phantom data. DXA scans were performed by a single radiographer. Appendicular skeletal muscle mass (ASM) was calculated from the sum of lean tissue mass for the arms and legs [4], and total fat mass was calculated by the sum of fat in the arms, legs, and trunk.

### 2.3. Physical Function Tests

Three physical performance tests were used to quantify physical function in this group. They included the six-minute walk (6 MW) test, the 9-item Physical Performance Test (PPT), and 4-meter gait speed.

 The 6 MW test is a measure of physical function and walking endurance. The participants walked back and forth in a hallway around, two which that were placed 25 meters apart. The participants were instructed that the goal of the assessment was to walk as far as possible in six minutes. The 6 MW test is a reliable performance-based measure of physical function in older adult populations that is related to overall locomotor ability [5, 6]. Six-minute walk distance was recorded as the total distance walked in meters and used for analysis.

 The modified 9-item PPT was used as a composite assessment of physical function. This test is designed to mimic activities of daily living and includes tests of standing balance, moving from sit to stand, light lifting, putting on and removing a jacket, walking, and stair climbing. The 9-item PPT has been shown to correlate well with disability and frailty [7]. Each item of the PPT is scored from 0 to 4, with the higher score indicating better performance. Total PPT score out of 36 possible was used for analysis.

 The four-meter gait speed test was performed over a 10-meter walking course. Participants were instructed to walk at their preferred walking pace and to continue walking over the entire 10-meter course. Timing took place over the middle four meters of the walking course. Gait speed is a reliable measurement that reflects health and functional status in older adults [8]. Gait speed was measured in meters per second.

### 2.4. Strength and Power Tests

Knee extension muscle strength (strength) and leg extension muscle power were quantified with a maximal isometric voluntary knee extension contraction test. Leg extension muscle power (power) was quantified by a test of concentric leg extension power.

 Isometric strength of the quadriceps femoris muscles of each lower extremity was evaluated at 60° of knee flexion on a dynamometer (Kin-Com 500 H, Chattecx Corp.; Harrison, TN). Following three practice trials, participants were tested three times with a two-minute rest between trials to avoid muscle fatigue. The strength measures, which were corrected for the influence of gravity, have demonstrated high reliability (ICC = 0.99) [9]. The average maximum force value in newtons, normalized to BMI was used for analysis.

 Leg extension power in each lower extremity was evaluated using the leg extension (Nottingham) Power Rig. Participants were positioned such that the hips were supported in a seat, which was adjusted to allow for 90 degrees of knee flexion in the starting position and ~5 degrees short of full knee extension in the finishing position. Following 4-5 practice trials, the participant performed 5 leg extension maximal efforts. The leg extension power rig is a feasible means of assessing muscle power across the lifespan [10]. The average maximal effort leg extension power in watts, normalized to BMI was used for analysis.

### 2.5. Statistical Analysis

Data management and statistical analyses were performed with PASW Statistics 18.0 (SPSS, Chicago, IL). Descriptive data were calculated for demographic variables and dependent measures and are presented as means ± SD. To determine the relationships between the two separate sarcopenia indices, Spearman's rank correlations were calculated between both absolute and relative sarcopenia and PPT, 6 MW, gait speed, strength, and power test scores. The Spearman's rank correlation coefficient is a nonparametric test of association between two variables [11]. This test was used instead of the Pearson's product moment correlation because of the nonnormality of distribution of sarcopenia indices and dependent measures. To determine how the relationship between absolute sarcopenia and the dependent measures was affected by total body fat, subjects were stratified, based on total body fat into equal tertiles, and regression coefficients were then calculated separately in each tertile. The alpha level was set at <0.05.

## 3. Results

The subjects included 202 (100 females and 102 males) nonobese community-dwelling older adult volunteers. Characteristics of the subjects are summarized in Table 1.

The prevalence of absolute sarcopenia in this group, based on the criteria of Baumgartner et al. [12], was defined as ASM/ht^2^ < 7.26 for males and <5.45 for females. Prevalence of absolute sarcopenia was 29.4% in the males and 28.0% in the females, with a range of 4.9–10.2 for males and 4.5–8.8 for females. The prevalence of relative sarcopenia, based on criteria established by Estrada et al. [3], which was defined as ASM/kg total body mass < 0.29 for males and <0.22 for females, was 42.2% in the males and 34.0% in the females, with a range of 0.23–0.40 for males and 0.18–0.33 in the females.

 Absolute sarcopenia (ASM/ht^2^) was not significantly associated with the PPT, 6 MW, or gait speed in males. Absolute sarcopenia was weakly related to 6 MW (*r* = 0.16, *P* = 0.02) and gait speed (*r* = 0.14, *P* = 0.05) in females. Relative sarcopenia (ASM/kg) was moderately associated with the PPT (*r* = 0.43, *P* < 0.001), 6 MW (*r* = 0.48, *P* < 0.001), and gait speed (*r* = 0.31, *P* < 0.01) in males, and with the PPT (*r* = 0.39, *P* < .001), 6 MW (*r* = 0.51, *P* < 0.001), and gait speed (*r* = 0.41, *P* < 0.001) in females. Knee extension strength and leg extension power were also not related to absolute sarcopenia in males but were related to absolute sarcopenia in females (*r* = 0.27, *r* = 0.41, resp., *P* < 0.001). Knee extension strength and leg extension power demonstrated significant relationships with relative sarcopenia in both sexes (*r* = 0.67, *r* = 0.66 in females; *r* = 0.47, *r* = 0.48 in males *P* < 0.001) (Table 2).

**Figure 1:**
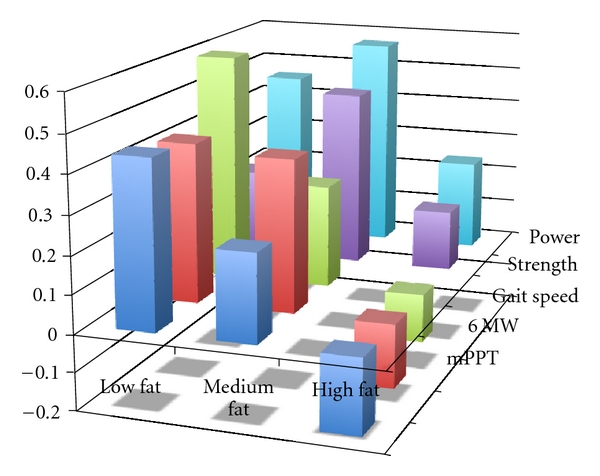
Standardized regression coefficients between absolute sarcopenia scores (independent variable) and mPPT score, 6-minute walk distance, gait speed, knee extensor strength, and lower extremity power (dependent variables) across equal low (34 males, 33 females), medium (34 males, 33 females), and high-fat (34 males, 34 females) tertiles.

To examine how the relationship between absolute sarcopenia and the dependent measures of PPT, 6 MW, gait speed, strength, and power were affected by total body fat, the subjects were stratified, by total body fat, into equal tertiles (Figure 1). This analysis revealed diminished regression coefficients across each incrementally higher fat grouping for the physical performance variables. In the low-fat group, the relationships between absolute sarcopenia and PPT (*r* = 0.44, *P* ≤ 0.001), and absolute sarcopenia and 6 MW (*r* = 0.42, *P* < 0.001), and absolute sarcopenia and gait speed (*r* = 0.60, *P* < 0.001) were higher than those in the medium fat (PPT *r* = 0.23, *P* = 0.07, 6 MW *r* = 0.40, *P* = 0.001, gait speed *r* = 0.27, *P* = 0.03), or high fat group (PPT *r* = −0.20  *P* = 0.11, 6 MW *r* = −0.17, *P* = 0.17, gait speed *r* = −0.13, *P* = 0.30). The analysis between absolute sarcopenia and both strength and power revealed generally higher regression coefficients for the low (strength *r* = 0.23, power *r* = 0.46, *P* < 0.001) and medium fat tertiles (strength *r* = 0.47, power *r* = 0.57, *P* < 0.001) and lower regression coefficients for the higher fat tertile (strength *r* = 0.16, *P* = 0.20 power *r* = 0.24, *P* < 0.05).

## 4. Discussion 

We completed a sarcopenia profile of 202 nonobese community-dwelling older adults. This allowed us to look at sarcopenia in both absolute (ASM/ht^2^) and relative (ASM/kg) terms. Consistent with previous literature [3, 13], the prevalence of sarcopenia increases when reported in relative versus absolute terms. The novel findings from this study are that relative sarcopenia was moderately related to a 9-item composite measure of actual physical performance, to the distance walked in six minutes and also to preferred gait speed in males and females. While we identified weaker, but significant relationships between absolute sarcopenia and 6 MW and between absolute sarcopenia and gait speed in females, no relationship existed between functional performance and absolute sarcopenia in the males. Other authors have suggested that relative sarcopenia seems to better capture the relationship between lean mass and physical performance [3, 14–16]; however, our data goes further, suggesting that total body fat modulates the relationship between sarcopenia and functional performance in nonobese older adults. There was a clear trend toward diminished regression coefficients across each incrementally higher fat grouping for the physical performance variables. These findings contribute to the rapidly growing trend considering sarcopenia relative to total body mass, and not simply total lean mass. 

 By stratifying the individuals in this study into tertiles of total body fat, our data clarifies that as total body fat increases, there is a diminished relationship between the independent variable of absolute sarcopenia with actual physical performance (PPT, 6 MW, and gait speed). Moreover, it appears that in older adults with higher body fat, absolute sarcopenia does not have as strong a relationship to muscle function (strength and power) as it does in older adults with medium-to-lower amounts of total body fat, suggesting that there may be a total body fat threshold at or over which may interfere with the relationship between lean tissue and functional performance. 

 The findings from this study introduce a plausible explanation why other studies have failed to demonstrate a relationship between sarcopenia and function, and why they have concluded that lean tissue mass is not linked to physical function in older adults. Our results suggest that in nonobese older adults who have lower total body fat relative to nonobese older adults who have higher total body fat, lean tissue mass may in fact be an important variable that influences physical function. Excess fat deposition may also contribute to a loss of lean tissue via inflammatory mechanisms, as cytokines have been shown to have direct catabolic effects on muscle [17]. Clinical support of this assertion is found in a recent paper by Koster et al. [18], who reported in nonobese older adults that although greater body fat mass was related to greater leg lean mass at baseline, body fat mass was also related to a significantly greater loss of leg lean mass over a seven-year follow-up. 

 An alternate way of interpreting our data is that even in a group of older adults who are not considered obese by BMI measurement, total body fat impacts physical function. This may be explained, in part, by the suggestion that BMI is not the best indicator of total body fat content in the older adult population and may remain stable in the presence of significant body composition alterations [19]. For example, a loss of muscle mass may go unnoticed in older adults who maintain or even gain fat mass. Middle-aged adults with normal BMI values but excess total body fat have recently been identified as normal weight obese (NWO). Just as individuals falling into this NWO category have been shown to be at higher cardiovascular disease risk [20], it may be that a similar category exists in older adults with parallel relationships with physical function. The stronger relationships observed between relative sarcopenia and physical function in this study may be due, in part, to the extra load carried by the participants in the higher fat tertiles. The physical performance consequence of excess fat deposition is in part substantiated by recently reported relationships between ectopic fat deposits in locomotor skeletal muscle (intramuscular adipose tissue) and muscle strength and quality [21, 22], mobility [23, 24], physical activity [25], and disability [26] in older adults. The direct influence of intramuscular adipose tissue on muscle function in this population should be further investigated. This may be especially important considering the recent trends of increased sedentary time and decreased physical activity with advancing age and the impact of these trends on adipose depositions, loss of muscle mass, and decreased physical function. 

 The results of this study should be considered in light of several limitations. The older adults in this investigation were for the most part high functioning individuals who fell into a relatively narrow BMI range with a mean BMI less than 27.0 kg/m^2^. In particular, it should be noted that individuals with the lowest levels of mobility function, that is, unable to independently ambulate, were not included in this sample. These results, therefore, cannot be generalized to all older adults. Ultimately, the clinical utility of these relationships will become apparent by determining whether interventions aimed at reducing fat deposition and increasing lean body mass in nonobese older adults result in improved physical function. Though this study was not designed to answer this question, future investigations should determine the impact of interventions on changing both body composition and physical function in this population. An additional limitation is that we were unable to define a relative sarcopenia cut-off for males and used a best estimate based on the available literature. However, we do not think this limitation detracts from our findings, as this was not the primary purpose of our investigation. 

 In nonobese older adults, total body fat modulates the relationship between sarcopenia and physical function. In nonobese older adults who have low amounts of total body fat, lean tissue appears to take on more clinical importance. While decreasing overall body fat even in those who are not traditionally considered obese appears warranted, frail individuals who have low BMI may specifically benefit from interventions aimed at increasing lean tissue mass. This point has recently been overshadowed by the most recent data characterizing lean mass as less important to physical function in older adults. 

## Figures and Tables

**Table 1:** Characteristics of the 202 participants (males *n* = 102, females *n* = 100).

Variable	Males	Females
	mean (SD)	mean (SD)
Age (years)	73.6 (5.8)	74.0 (6.1)
Body mass index (kg* m^2^)	26.6 (3.8)	26.6 (4.5)
Total fat (kg)	21.8 (7.8)	26.2 (8.9)
Total lean (kg)	53.2 (7.5)	36.4 (6.0)
Physical performance test (36 possible)	31.8 (4.4)	30.3 (4.7)
Knee extension strength (*N*)	384.5 (101.4)	247.0 (78.2)
6-minute walk (meters)	512.0 (109.5)	445.7 (106.6)
Self-selected gait speed (m/s)*	1.52 (0.26)	1.36 (0.29)

*gait speed calculated by 4 meter gait speed test.

**Table 2:** Bivariate Correlation Results. Absolute (ASM/Ht^2^) and relative (ASM/kg) sarcopenia and modified physical performance test (mPPT), six-minute walk (6 MW), gait speed, normalized knee extension strength (*N*/BMI), and normalized leg extensor power (*W*/BMI).

	Absolute sarcopenia				Relative sarcopenia			
	Males	*P*	Females	*P*	Males	*P*	Females	*P*
mPPT	0.03	0.77	0.07	0.36	0.43	<0.001	0.39	<0.001
6 MW	0.03	0.73	0.16	0.02	0.48	<0.001	0.51	<0.001
Gait speed	0.04	0.67	0.14	0.05	0.31	0.002	0.41	<0.001
Knee ext strength	−0.04	0.69	0.27	<0.001	0.47	<0.001	0.67	<0.001
Leg ext power	0.16	0.10	0.41	<0.001	0.48	<0.001	0.66	<0.001
